# Knowledge of symptoms and delays in diagnosis of extrapulmonary tuberculosis patients in North Shewa zone, Ethiopia

**DOI:** 10.1371/journal.pone.0270002

**Published:** 2022-06-16

**Authors:** Awraris Hailu Bilchut, Alemayehu Gonie Mekonnen, Tigist Abetew Assen

**Affiliations:** 1 School of Public Health, Asrat Woldeyes Health Science Campus, Debre Berhan University, Debre Berhan, Amara Regional State, Ethiopia; 2 School of Nursing and Midwifery, Asrat Woldeyes Health Science Campus, Debre Berhan University, Debre Berhan, Amara Regional State, Ethiopia; The University of Georgia, UNITED STATES

## Abstract

**Introduction:**

Delayed diagnosis contributes to the high burden and transmission of tuberculosis and extrapulmonary tuberculosis (EPTB) and continued to be a major public health problem in Ethiopia. Currently, there is insufficient knowledge on the contributing factors to diagnostic delay of EPTB patients in healthcare settings in Ethiopia, because of unique cultural and societal issues in this country. This study assessed patients’ knowledge of symptoms and contributing factors of delay in diagnosis of EPTB patients at selected public health facilities in North Shewa zone, Ethiopia.

**Methods:**

An institutional-based study was conducted from March to April 2021. All recently registered EPTB patients were included. Logistic regression was performed to analyze the data. A significant association was declared at a p-value of < 0.05, and the results were presented with an adjusted odds ratio (AOR) and the corresponding 95% confidence interval (CI).

**Results:**

In this study, only 15.5% of respondents knew EPTB symptoms. The median patient and healthcare system delay was 55 days. A patient delay of greater than 3 weeks and a health system delay of greater than 2 weeks were observed among 85.2% and 81% of patients, respectively. After the end of 5 weeks, 87.3% of EPTB patients had been diagnosed with the disease and the total median delay was 108.5 days. Living more than ten kilometers far from a health facility (AOR = 1.54; 95% CI = 1.11, 4.63), having never heard of EPTB disease (AOR = 5.52; 95% CI = 1.73, 17.56), and having ever taken antibiotics at the first health facility visit (AOR = 7.62; 95% CI = 2.26, 25.65) were associated with a total diagnostic delay of beyond 5 weeks.

**Conclusions:**

The diagnostic delays of EPTB remain high. Both patient and health system delays equally contributed to the total diagnosis delay. Improving community awareness of EPTB and advancing diagnostic efficiencies of healthcare facilities could help reduce both delays.

## Introduction

Tuberculosis (TB) is an airborne, contagious disease caused by bacteria called *Mycobacterium tuberculosis* [1, 2]. The bacteria commonly affects the lung and its symptoms include a cough that lasts for more than 2 weeks, weight loss, night sweats and loss of appetite. It also attacks other parts of the body such as the intestine, genitourinary tract, bone, kidney and lymph nodes [3, 4]. If the bacteria affect any part of the body other than lung parenchyma, it is referred to as extrapulmonary tuberculosis (EPTB) [4, 5]. Patients with EPTB have enlarged lymph nodes and musculoskeletal pain and they also complain of other types of non-specific symptoms [6–8].

Delayed medical consultation contributed to the high burden and transmission of tuberculosis and EPTB continued to be a major public health problem in Ethiopia [9]. The country was one of the Sub-Saharan countries that reported the highest number of new tuberculosis patients and including EPTB cases [4, 10, 11]. Since patients with EPTB commonly have vague clinical presentations as compared to pulmonary TB patients, timely detection of EPTB is challenging and the patients rarely seek medical attention which further contributes to the diagnostic delay of the illness [12]. Delay in diagnosising and treating EPTB significantly reduce disease prognoses at the individual level and affects the healthcare systems at large [13]. The healthcare facilities become loaded with more complicated TB diseases which results in extra healthcare costs to treat complex disease forms [14]. On the other hand, timely detection and initiation of anti-TB treatment decrease disease severity and economic costs for the patient and their families [15]. It also reduces mortality and morbidity as well as improves the efficiency and effectiveness of TB control efforts [16, 17].

Despite the advance in diagnosis and expansion of TB services, patients’ poor health-seeking behaviour and diagnostic delay of EPTB cases remained problematic in Ethiopia and the case-detection efforts are unsuccessful and TB is very widespread in the country [18, 19]. Many community-based studies reported that the number of undetected EPTB cases in communities can be greater than the number of notified cases [20, 21]. For example, out of the estimated EPTB cases, only a few cases were notified to the national tuberculosis program in 2017. EPTB was also associated with a longer delay in treatmrnt [22]. The problem is more prominent in areas where there is poor healthcare system infrastructure and limited healthcare facilities such as the North Shewa zone.

The previous studies have described diagnostic delays and associated factors among patients with pulmonary tuberculosis. But, there is insufficient knowledge on the pattern and the contributing factors of diagnostic delay of EPTB patients in different healthcare settings in Ethiopia, because of unique cultural and societal issues in this country. It is important to find out specific factors that contribute to delay in diagnosis in order to enhance case detection and treatment. This study, therefore, assessed patients’ knowledge of symptoms and contributing factors in diagnosis of EPTB patients at selected public health facilities in the North Shewa zone, Ethiopia.

## Methods

### Study design, setting and period

An institutional-based cross-sectional study was conducted from March 1, 2021 to April 30, 2021 in 15 health facilities in the North-Shewa zone, Amhara regional state, Ethiopia. The zone has 24 administrative districts with an estimated population of more than 2 million. There are currently 10 hospitals, 97 health centers and 389 health posts in the North-Shewa zone. EPTB diagnosis and treatment services, including direct-observed-treatment, are now available in all hospitals and health centers. Health extension workers who are employed in health posts play an important role in detecting and referring TB suspects to the next level of healthcare facilities for diagnosis and initiation of anti-TB treatment. Fifteen health facilities were selected from Debre-Berhan, Minjar-Shenkora, Menz-mama, Menz-Gera, Shewrobit, Efratana-Gidim, Siyadebir, Angolela and Kewot districts as these health facilities had an improved referral linkage of TB treatment with hospitals.

### Respondents and sampling procedures

The study participants were EPTB patients who followed up their anti-TB treatment in 15 public health facilities of Debre-Berhan, Minjar-Shenkora, Menz-mama, Menz-Gera, Shewrobit, Efratana-Gidim, Siyadebir, Angolela and Kewot districts. All newly registered EPTB patients who were in the intense phase of therapy were included. Over the course of 2 months, these EPTB patients were approached for interviews.

### Measurements

Patient delay was assessed by asking patients to recall the duration of time in a day from the perceived onset of symptom(s) of the illness to their first contact with a health facility. Health system delay was assessed by asking patients to recall the duration of time in days from their first visit to a health facility to the date of EPTB diagnosis. Patients’ knowledge of EPTB was assessed by 3 categories. These include: *Have you ever heard about EPTB diseases*? *What are the ways of EPTB transmission*? *What are the symptoms of EPTB*? Respondents could answer either “Yes” or “No” from the listed options. *“Yes”* answers were considered as correct and *“No”* were considered incorrect. Mean scores were calculated to classify the respondents into 2 groups (knowledgeable and not knowledgeable about ways of EPTB transmission and its symptoms). Respondents who answered above the mean score were classified as knowledgeable, and the rest was classified as not knowledgeable.

### Operational definitions

#### Patient delay

is the duration from the onset of the first symptoms (cough, fever, night sweat, loss of appetite and muscle pain) to the first visit to a health facility. The time longer than 3 weeks was considered a patient delay.

#### Health system delay

is the time interval between the first visit to the health facility and the date of EPTB confirmation. The time longer than 2 weeks was considered a health system delay.

#### Total delay

is the time interval between the onset of the first symptoms and the date of EPTB confirmation. It is the sum of both patient delay and health system delay. The time longer than a value of 5 weeks was assumed as a total delay [16].

### Data collection

An interviewer-administered questionnaire was used to collect the data. The questionnaire was pre-tested and designed first in English and then translated into Amharic (native language). The questionnaire was adapted from both TB Care-II guidelines and previous literature [15, 17, 23]. Exit interviews were applied by trained enumerators. Fifteen diploma nurses who worked in TB clinic participated in the data collection. Data completeness was checked by the investigators.

### Data processing and analysis

Epi-data version 3.2 software was used for data entry and data were exported to SPSS version 21. Descriptive statistics were computed to explore the data. Logistic regression was performed to analyze the data. Logistic regression analyses were performed between the independent and the outcome variables. Those independent variables which were statistically significant in the bivariate model (*p-value <0*.*25*) were entered into the multivariable analysis. In the final model, a significant association was declared at a p-value of less than 0.05, and the results were presented with an adjusted odds ratio (AOR) and 95% confidence interval (CI).

### Ethical considerations

Ethical approval was obtained from the research and ethical review committee of Debre Berhan University. Written informed consent was obtained from each study participant and assent was obtained from parents of children under 18 years old. All the information obtained from participants was kept confidential throughout the process of study, and the name of the participant was replaced by a code. Withdrawal from the study at any point if they wished was assured.

## Results

### Socio-demographic characteristics

In this study, 142 EPTB patients were were interviewed, with a response rate of 98.6%. The mean age of the respondents was 41.4(±15.8 SD) years. More than half of the respondents were female (52.8%) and 54.9% of participants were married. Nearly one-third (32.4%) of study participants were a farmer and 41.5% of respondents were not educated. In terms of access to healthcare facilities, 64.8% of respondents lived far away, about 10 kilometers, from health facility (Table 1).

**Table 1:** Sociodemographic characteristics of EPTB patients at public health facilities of North Shewa zone, Ethiopia, April 2021.

Variables	Categories	N (%)
Age in years	≤24	16 (11.2)
	25–34	33(23.2)
	35–44	35(24.6)
	45–54	21(14.8)
	>55	37(26.1)
Gender	Male	67(47.2)
	Female	75(52.8)
Place of residency	Rural	82(57.7)
	Urban	60(42.3)
Marital status	Married	78(54.9)
	Unmarried	31(21.8)
	Divorced/widowed	33(23.2)
Educational status	No education	59(41.5)
	Primary	50(35.2)
	Secondary	23(16.2)
	College and above	10(7.0)
Occupational status	Employed	43(30.3)
	Housewife	29(20.4)
	Farmer	46(32.4)
	Other (student, daily labour)	24(16.9)
Distance to the nearest health facility	<10 kilometers	50(35.2)
	≥10 kilometers	92(64.8)

### Clinical characteristics of study participants

As shown in Table 2, 30% of respondents reported musculoskeletal pain, and twenty percent reported an enlarged lymph node. Approximately three-quarters of the respondents (63.4%) had taken antibiotics during their first visit to the health facility. One-third (33.8%) of the EPTB cases were co-infected with the human immunodeficiency virus (HIV) (Table 2).

**Table 2:** Clinical characteristics of EPTB patients at public health facilities of North Shewa zone, Ethiopia, April 2021.

Variables	Categories	N(%)
The first symptom develop	Night sweat	19(13.4)
	Loss of appetite	27(19.0)
	Weight loss	10(7.0)
	Enlarged lymph node	29(20.4)
	Musculoskeletal pain	43(30.3)
	Cough >2wks	14(9.9)
Took antibiotic at first health facility visit	Yes	90(63.4)
	No	52(36.6)
HIV status	Negative	94(66.2)
	Positive	48(33.8)

### Knowledge of participants about EPTB

Of the total study participants, only 28.9% of respondents ever heard about EPTB diseases.

One-third of the participants (33.1%) were aware of the routes of EPTB transmission, but only 15% were aware of the symptoms. Nearly half of the respondents (47.9%) thought that EPTB could be cured. Regarding the health-seeking practice, more than half of the respondents (66.2%) sought healthcare professionals when they felt EPTB symptoms (Table 3).

**Table 3:** Respondents’ knowledge of EPTB at public health facilities of North Shewa zone, Ethiopia, April 2021.

Variables	Yes	No
Have you ever heard about EPTB diseases?	41(28.9)	101(71.1)
Do you think that EPTB is a serious disease?	45(31.7)	97(68.3)
**What are the ways of EPTB transmission?**		
Coughing and sneezing of TB patients	35(24.6)	107(75.4)
Droplet infection and direct contact with TB patient	88(62.0)	54(38.0)
Do not know	19(13.4)	
**Average knowledge of EPTB transmissions**	**47(33.1)**	**95(66.9)**
**What are the symptoms of EPTB?**		
Night sweat	15(10.6)	127(89.4)
Loss of appetite	22(15.5)	120(84.5)
Weight loss	23(16.2)	119(83.8)
Enlarged lymph node	12(8.5)	130(91.5)
Cough >2wks	29(20.4)	113(79.6)
Musculoskeletal pain	30(21.1)	112(78.9)
Do not know	21(14.8)	
**Average knowledge of EPTB symptoms**	**22(15.5)**	**120(84.5)**
Have you ever visited a health facility when you felt EPTB symptoms?	94(66.2)	48(33.8)
Do you think that EPTB patients can be cured?	68(47.9)	74(52.1)

### Delays in EPTB diagnosis

**Fig 1:**
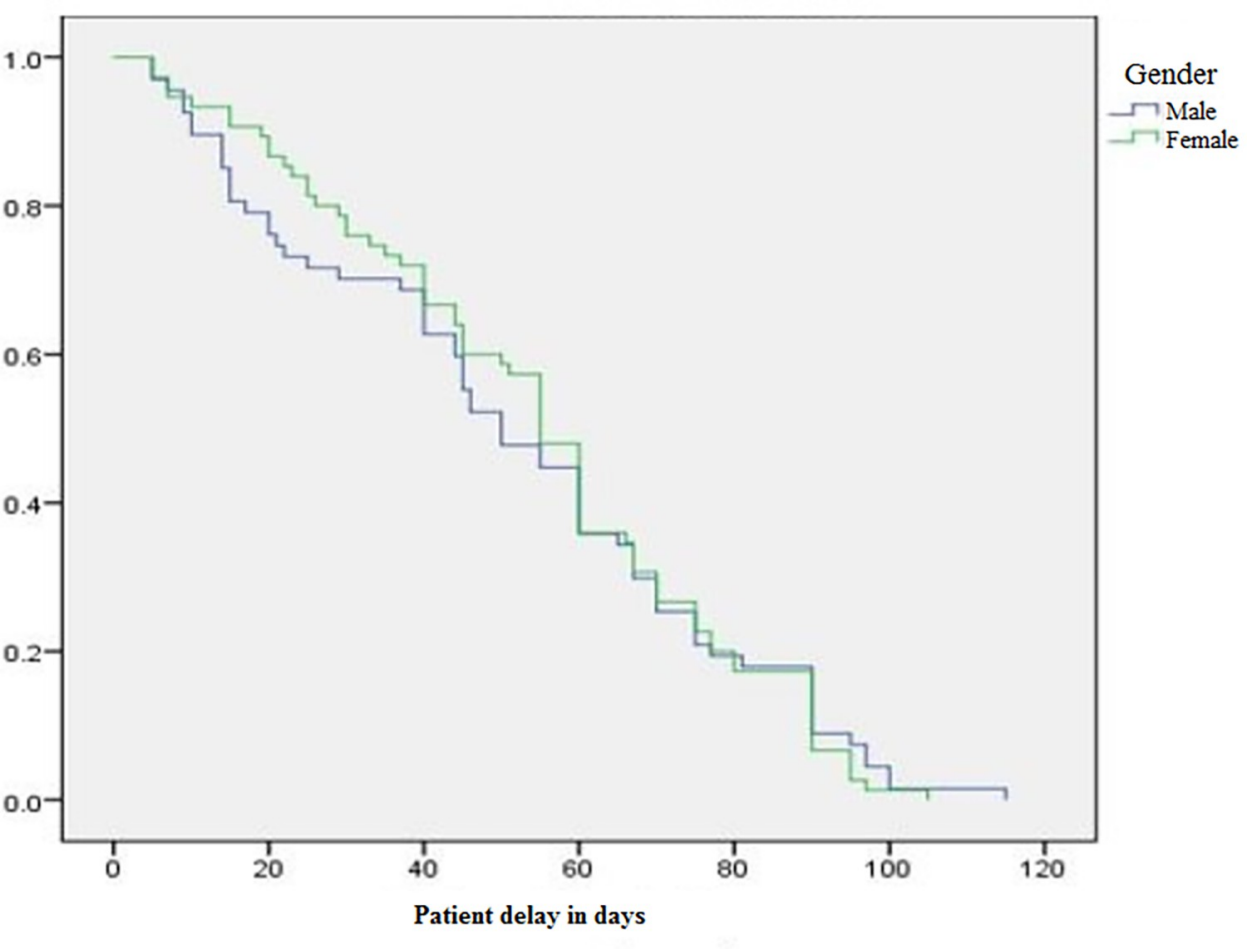
Kaplan–Meier curve of patient delays by gender of respondents.

**Fig 2:**
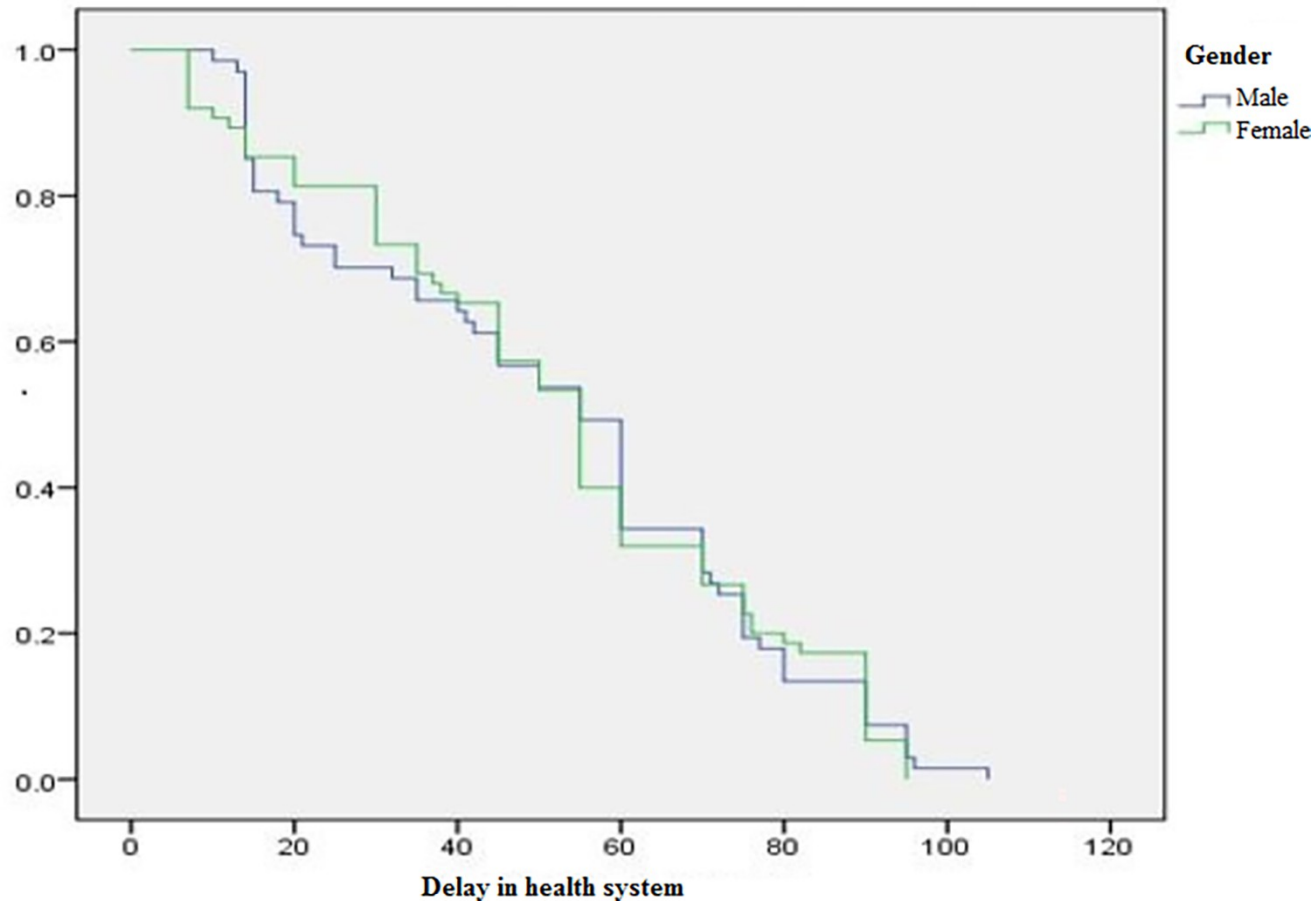
Kaplan–Meier curve of health system delays by gender of study participants.

Even though the range varies, the median patient and healthcare system delay was the same (55 days). A patient delay of greater than 3 weeks and a health system delay of greater than 2 weeks were observed among 85.2% (CI = 78.6, 90.1) and 81% (CI = 74.8, 87.3) of patients, respectively. After the end of 5 weeks (i.e. the cut-off point for the total diagnostic delay), 87.3% (CI = 81.4, 92.3) of EPTB patients had been diagnosed with the disease and the total median delay was 108.5 days (range 19–195 days) (Table 4). Figs 1 and 2 show the Kaplan–Meier curve of patient and health system delays by gender of the study participants.

**Table 4:** Delay status of EPTB patients at public health facilities of North Shewa zone, Ethiopia, April 2021.

Patients delays	N(%)	95%CI
≤3 weeks	27(19.0)	(12.7, 25.2)
>3 weeks	115(81.0)	(74.8, 87.3)
**Median patient delay in days (range)**	**55 (5–115)**	
**Healthcare system delays**		
≤2 weeks	21(14.8)	(9.9, 21.4)
>2 weeks	121(85.2)	(78.6, 90.1)
**Median healthcare system delay in days (range)**	**55(7–105)**	
**Total/diagnostic delays**		
≤5 weeks	18(12.7)	(7.7, 18.6)
>5 weeks	124(87.3)	(81.4, 92.3)
**The median total delay in days (range)**	**108.5(19–195)**	

### Patients’ reasons for delays in seeking EPTB treatment

**Fig 3:**
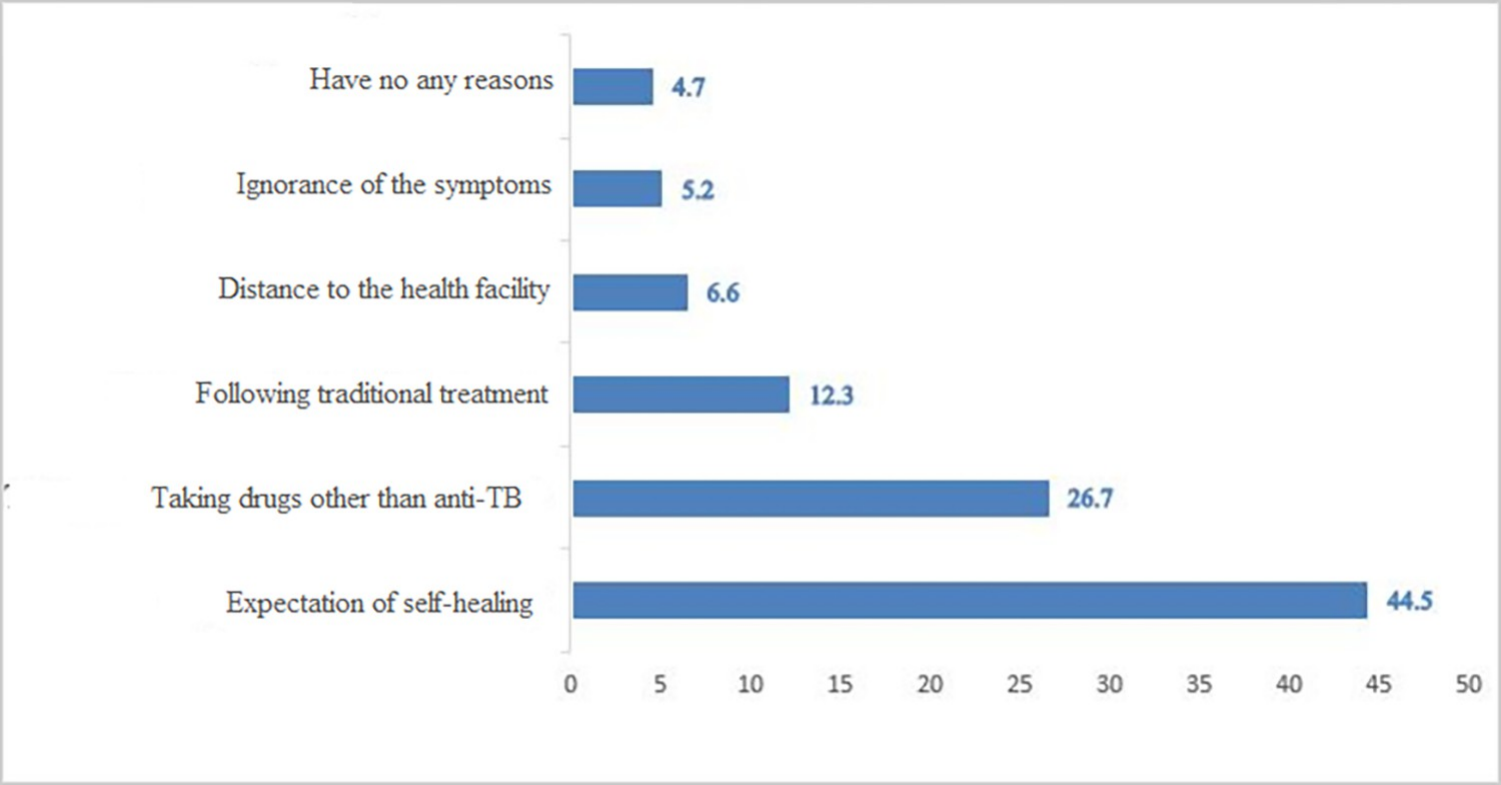
Participants’ reason for delays of EPTB diagnosis at public health facilities of North Shewa zone, Ethiopia, April 2021.

Study participants were asked about the reason why they delayed in seeking EPTB treatment. The anticipation of self-healing (44.5%), obtaining antibiotics other than anti-TB from health facilities (26.7%), and self-treatment with traditional medicine (12.3%) were the most common reasons given by study participants (Fig 3).

### Determinants of delays in EPTB diagnosis

In the multivariable analysis, there was no significant association between the independent variable and the exposure variables of patient and health system delay. However, the total diagnostic delay was associated with the distance of the health facility, prior awareness of EPTB disease and antibiotic use. Living more than 10 kilometers far from a health facility (AOR = 1.54; 95% CI = 1.11, 4.63), having never heard of EPTB disease (AOR = 5.52; 95% CI = 1.73, 17.56), and having ever taken antibiotics at the first health facility visit (AOR = 7.62; 95% CI = 2.26, 25.65) were associated with a total diagnostic delay of beyond five weeks. Those patients had a higher odds ratio as compared to their counterparts (Table 5).

**Table 5:** Analysis of factors associated with diagnostic delay of EPTB patients at public health facilities of North Shewa zone, Ethiopia, April 2021.

Variables	Category	Total delay		COR (95%CI)	AOR (95%CI)
		≤5 weeks	>5 weeks		
Distance to the nearest health facility	<10 kilometers	9	41	1	1
	≥10 kilometers	9	83	2.02(1.75, 5.45)	1.54(1.11, 4.63)*
Ever heard about EPTB disease?	Yes	10	31	1	1
	No	8	93	3.75(1.36,10.34)	5.52(1.73,17.56)*
Ever took antibiotics at first health facility visit	Yes	5	85	5.66(1.88,17.01)	7.62(2.26,25.65)*
	No	13	39	1	1

1 = reference

* p-value <0.05

## Discussion

Basic information about TB symptoms, transmission and treatment options help patients seek medical consultation at health facility which in turn reduce diagnostic and treatment delay in TB cases [24]. In this study, One-third of the respondents were aware of the routes of EPTB transmission, and only fifteen percent were aware of the symptoms; this finding is consistent with a study in India [12]. This low level of knowledge of EPTB symptoms could probably be explained by a lack of information about EPTB disease and vague clinical symptoms of EPTB patients. These symptoms are sometimes missed by trained healthcare workers, which can contribute to both patients and health systems delays [24]. This finding underscores the need for improved community knowledge of EPTB, which can lead to eary initiation of the treatment.

Despite the lack of a well-defined cut-off point for acceptable EPTB diagnosis delays, 85.2% and 81% of EPTB patients, experienced patient delays of more than 3 weeks and health system delays of more than two weeks, respectively. After the end of 5 weeks, 87.3% of EPTB patients had been diagnosed with the disease and the total median delay was 108.5 days. This figure was greater than prior reports in Zanzibar [15], as well as the reported delay in pulmonary tuberculosis cases in Addis Ababa [24] and Bahrdar city [9, 24]. Surprisingly, the reported delays in our study are higher than those reported in Ethiopian pastoralist communities [10, 18]. This is probably because of patients’ poor knowledge of EPTB symptoms which in turn affect their health-seeking behaviour. Poor health system structure like shortage of laboratory facilities and geographical factors may also contribute to the long delays of EPTB diagnosis. Hence, health professionals alone may have a limited ability to solve both patient and health system delays. Instead, more coordinated efforts by all those involved in TB control programs including the governments might be required to avoid severe delays in EPTB diagnosis.

Both patient and health-care system delays contributed equally to the total diagnosis delay in our study. Previous research, however, had mixed results. In previous studies, for example, frequent visits to the same healthcare level were associated with a longer health system delay [16, 25, 26]. This is most likely attributable to differences in healthcare system efficiency and the study population’s characteristics in the different study contexts.

In our study, living more than 10 kilometers far from the nearest health facility, having never heard of EPTB disease, and having ever taken antibiotics at the first health facility visit were associated with higher odds of patient delay. Patients who lived more than 10 kilometers away from a health institution were more likely to delay more than 5 weeks in getting an EPTB diagnosis. Similar studies also reported the association between long-distance and delay of EPTB diagnosis [9, 27]. This implies that limited accessibility of healthcare facilities could affect the implementation of TB programs and could delay the diagnosis of potentially infectious pulmonary TB [28, 29]. Our data also revealed that patients who never heard about EPTB disease were more likely to delay longer than 5 weeks. This suggests that a lack of awareness about EPTB disease may be related to poor medical seeking behavior.

The existing evidence suggested that inappropriate prescription of antibiotics is associated with increased self-healing behaviour of the patient [28]. This evidence was supported by our study in which EPTB patients who have ever taken antibiotics at the first health facility visit were more likely to delay beyond 5 weeks. Ever using non-anti-TB medications as a factor in diagnosis delay was also reported in the previous study [3, 14]. This is due to the fact that healthcare providers commonly encounter limitations in detecting EPTB as those patients with EPTB frequently had unclear clinical presentations, and they often gave antibiotics to the patient which further contributes to the diagnostic delay of the illness. On the other hand, if the patients were given an antibiotic at the first health facility contact, they may have hoped for gradual healing and did not seek medical help unless severe complications were manifested.

### Limitations of the study

Despite its strength, this study has some limitations. Reports from a small sample size may be less reliable. There could also be recall bias in recognizing the first symptom and the date of the first visit to the health facility, as well as the diagnosis confirmation. Since there is no standardized definition of delays in EPTB treatment, the results of this study should be interpreted cautiously.

## Conclusion

In this study, the diagnostic delays of EPTB remain high. Both patient and health system delays equally contributed to the total diagnosis delay. Improving community awareness of EPTB and advancing diagnostic efficiencies of healthcare facilities could help reduce both patient and health system delays.

## Supporting information

S1 File English version of the questionnaire and consent form. (PDF)

## Abbreviations

AOR: Adjusted Odds Ratio
COR: Crude Odds Ratio
CI: Confidence Interval
EPTB: Extra Pulmonary Tuberculosis
HIV: Human Immunodeficiency Virus
TB: Tuberculosis
