# Supplementary material for: Knowledge of symptoms and delays in diagnosis of extrapulmonary tuberculosis patients in North Shewa zone, Ethiopia
Source: PLoS One. 2022 Jun 16;17(6):e0270002. doi: 10.1371/journal.pone.0270002 (PMC9202887; doi:10.1371/journal.pone.0270002)
Supplement: S1 File — (PDF) [file pone.0270002.s001.pdf]

English version of the questioner and consent form

Consent form

Dear Madam ----------------------------

My name is ----------- and currently working at Debre Berhan university. The aim of this study is assessing Knowledge of symptoms and delay in diagnosis of extra pulmonary tuberculosis patients in North Shewa zone, Ethiopia. Findings from this research help health care provider to have information EPTB. This study will also enable to advise policy makers on strategies to minimize this group's problems.

Thus this interview is prepared for this purpose to get the right information on the topic. The information that will be obtained using this interview will be used only for research purpose. Confidentiality and anonymity is fully assured, as your name is not required and only the research team will have access to the results. It will not affect you in anyway.

Should you not take part in this study? If **yes,** continue to sign consent form

If No, stop here

I have been informed that the purpose of this study is assessing patients' knowledge of symptoms and contributing factors of delay in diagnosis of extra pulmonary tuberculosis patients at selected public health facilities in the North Shewa zone, Ethiopia. I have understood that participation in this study is entirely voluntarily. I have been told that my answer to the question will not be given to anyone else and no reports of this study identify me in any way. I understood that participation in this study does not involve risks. I understood that Alemayehu Gonie is a contact person if I have question about the study or about my right as a study participant.

Respondent's Signature ___________Date___________

Start interview

Supervisor's name ________________ signature _________

English language version of the questioner

Instruction to the interviewer: circle the number in front of the option based on the response.

**Part I: Demographic Characteristics**	**Options**	**Remark**
Age in years	________________	
Gender	Male	
	Female	
Place of residency	Rural	
	Urban	
Marital status	Married	
	Unmarried	
	Divorced/widowed	
Educational status	No education	
	Primary	
	Secondary	
	College and above	
Occupational status	Employed	
	Housewife	
	Farmer	
	Other (student, daily labour)	
Distance to the nearest health facility	<10 kilometers	
	≥10 kilometers	
The first symptom develop	Night sweat	
	Loss of appetite	
	Weight loss	
	Enlarged lymph node	
	Musculoskeletal pain	
	Cough >2wks	
Took antibiotic at first health facility visit	Yes	
	No	

HIV status	Negative	
	Positive	
**Part II: assessment of respondents' knowledge on EPTB**	**Yes**	**No**
Have you ever heard about EPTB diseases?		
Do you think that EPTB is a serious disease?		
**What are the ways of EPTB transmission?**		
Coughing and sneezing of TB patients		
Droplet infection and direct contact with TB patient		
Do not know		
**What are the symptoms of EPTB?**		
Night sweat		
Loss of appetite		
Weight loss		
Enlarged lymph node		
Cough >2wks		
Musculoskeletal pain		
Do not know		
Have you ever visited a health facility when you felt EPTB symptoms?		
Do you think that EPTB patients can be cured?		
**Part III: Delay status of EPTB patients**		

What was the duration from the onset of the first symptoms (cough, fever, night sweat, loss of appetite and muscle pain) to the first visit to a health facility? In days.		
What as the time interval between the first visit to the health facility and the date of EPTB confirmation? In days		
What was the time interval between the onset of the first symptoms and the date of EPTB confirmation? In days		
What was your reason for delays in seeking EPTB treatment?<br><br>Probe		
**Thank you for your participation!!!!**		
